# Associations of childhood maltreatment with pre-pregnancy obesity and maternal postpartum mental health: a cross-sectional study

**DOI:** 10.1186/s12884-017-1565-4

**Published:** 2017-11-22

**Authors:** Michaela Nagl, Franziska Lehnig, Holger Stepan, Birgit Wagner, Anette Kersting

**Affiliations:** 1 Leipzig University Medical Center, IFB AdiposityDiseases, Philipp-Rosenthal-Str. 27, 04103 Leipzig, Germany; 2 Department of Psychosomatic Medicine and Psychotherapy, University of Leipzig, Semmelweissstr. 10, 04103 Leipzig, Germany; 3 Department of Obstetrics, University of Leipzig, Liebigstr. 20a, 04103 Leipzig, Germany; 4 Department of Clinical Psychology and Psychotherapy, MSB Medical School Berlin, Calandrellistraße 1-9, 12247 Berlin, Germany

**Keywords:** Childhood maltreatment, Body mass index, Obesity, Pregnancy, Depression, Anxiety

## Abstract

**Background:**

Pre-pregnancy obesity and postpartum mental disorders are prevalent health risks to both the mother and the offspring. The objective of our study was to examine whether a history of childhood maltreatment is associated with pre-pregnancy obesity and postpartum mental health and whether childhood maltreatment and pre-pregnancy BMI independently predict postpartum mental health.

**Methods:**

We obtained self-reported data from 741 postpartum women before 16 weeks after delivery (M = 8.1 weeks, SD = 3.2). Childhood sexual, physical, and emotional abuse and physical and emotional neglect were assessed with the Childhood Trauma Questionnaire. Depression and anxiety were assessed using the BDI and SCL-90-R. We conducted logistic regression models adjusted for demographic covariates and co-occurrence of different types of maltreatment.

**Results:**

7.6% of the included women entered pregnancy with obesity. Forty-six percent reported any type of childhood maltreatment. 6.4% displayed at least moderate postnatal depressive symptomatology and 20.5% scored above the 75th percentile for postpartum anxiety. Severe physical abuse, moderate emotional abuse and severe physical and emotional neglect were associated with pre-pregnancy obesity. After controlling for the presence of all other types of childhood maltreatment, only severe physical abuse was still predictive for pre-pregnancy obesity (adj.OR = 5.24, 95% CI = 1.15-23.75). Pre-pregnancy obesity was associated with an increased risk of postpartum depression (adj.OR = 2.55, 95% CI = 1.08-6.00) but not with elevated anxiety. Pre-pregnancy obesity and severe childhood sexual abuse independently predicted postpartum depression. After controlling for histories of different types of childhood maltreatment, the association between pre-pregnancy obesity and postpartum depression attenuated to non-significance.

**Conclusions:**

To our knowledge, this is the first study examining empirical relations between childhood maltreatment, pre-pregnancy BMI and postpartum mental health controlling for the co-occurrence of other maltreatment types. Childhood maltreatment has been found being associated with both pre-pregnancy obesity and impaired postpartum mental health and may at least partly account for the association between pre-pregnancy obesity and postpartum depression. Therefore childhood maltreatment is related to two common risk conditions during pregnancy and postpartum which bear several health risks for the mother and the child, and routine screening for histories of childhood maltreatment among pregnant women is warranted.

## Background

The globally rising prevalence of obesity makes maternal obesity one of the most common risk obstetric conditions. Applying the WHO criteria, the prevalence of maternal obesity varies from 1.8% to 25.3% [1]. Maternal obesity is a unique type of adult obesity due to its association with a variety of serious adverse health outcomes for the mother and the fetus [1–5]. Maternal perinatal complications include gestational diabetes, pregnancy-induced hypertension, pre-eclampsia [1, 3, 4], and cesarean delivery [1, 4, 5]. Fetal risks include miscarriage, stillbirth [1, 4, 6, 7], congenital anomalies [1, 3, 6], macrosomia, and childhood obesity [1, 3, 5, 8].

Mental disorders are common during pregnancy and postpartum. In a U.S. community-based survey among women with a past-year pregnancy the 12-month prevalence for psychiatric disorders was 25.3%. The 12-month prevalence for mood and anxiety disorders was 13% [9]. Studies conducted in non-pregnant adults provide evidence for a positive association between obesity and mental disorders, including depression [10–12] and anxiety [13]. Only few studies examined this relationship during pregnancy thus far. Overall, these studies also suggest a positive association [14–18]. Molyneaux et al. [16] estimated 43% higher odds of antenatal depression and 30% higher odds of postnatal depression among women who entered pregnancy with obesity compared to normal weight pregnant women. Furthermore, women with obesity had a 1.41-fold increased risk of antenatal anxiety. Maternal pre- and postnatal mental health have been found important factors influencing offspring cognitive and behavioral development as well as the interaction between mother and infant [19–22]. If untreated, the impairment of maternal mental health can result in negative outcomes for both the mother and the offspring [20].

Childhood maltreatment is common. In a German community-based representative study using the Childhood Trauma Questionnaire, the prevalence of childhood emotional, physical and sexual abuse was 15%, 12%, and 12.6%. 48.4% reported childhood physical neglect, 49.5% emotional neglect. There is evidence from general population studies, that childhood maltreatment is associated with a variety of adverse long-term physical and mental health outcomes [23], including obesity, depression and anxiety [24–29]. In particular, childhood maltreatment, due to a strong or prolonged activation of the body’s stress regulation system, may lead to a persistent alteration in neurophysiological systems which increases the risks of long-term adverse health outcomes [28]. Furthermore, there is evidence that individuals with a history of childhood maltreatment are at high risk of re-victimization in adulthood; e.g. to experience intimate partner violence or abuse in adulthood, which may lead to an aggravation of long-term health consequences [30]. Results from prospective general population studies suggest that childhood sexual and physical abuse are predictive of higher BMI gains from childhood into adulthood compared to non-abused individuals [31–33]. A recent meta-analysis revealed that childhood physical and sexual abuse increase the odds of depression in later life by 49 to 104% and those for anxiety by 70 to 152% [28]. Less is known about these associations during pregnancy. We are aware of only two studies with a focus on associations between different types of childhood maltreatment and obesity among pregnant women [34, 35] consistently suggesting an increased risk of pre-pregnancy obesity among women with a history of childhood physical abuse. Findings for emotional abuse are inconsistent with one study suggesting a positive association [34] and the other suggesting no association [35]. Particularly childhood abuse has also been found to be associated with ante- and postnatal depression, anxiety and generalized anxiety disorder among pregnant women [20, 30, 36–38].

Summarizing, we can conclude that childhood maltreatment has been found to be associated with both, maternal obesity and impaired pre- and postnatal mental health and therefore could increase the likelihood of either obesity or mental disorders during the pregnancy and postpartum. Furthermore, if childhood maltreatment is associated with both conditions, and occurs before the development of obesity and impaired mental health, childhood maltreatment could at least partly account for the association between maternal obesity and mental health during pregnancy and postpartum. To the best of our knowledge there is no study examining associations between all three factors among pregnant/postpartum women thus far.

Many studies examining associations between childhood maltreatment and adult obesity or mental health have included only one single form of childhood maltreatment (e.g., sexual abuse) or have examined the effects of different forms of childhood maltreatment separately [26, 36]. This method is likely to overestimate the influence of a single type of childhood maltreatment due to the common co-occurrence of other forms of childhood maltreatment [39]. Furthermore, many studies have compared individuals with and without obesity as a dichotomous category [26]. This might lead to an underestimation of the association of childhood maltreatment and obesity relative to normal weight as underweight has also been shown to be positively related to childhood maltreatment [40].

In our study, we aimed to take these drawbacks into account and to provide a detailed picture of associations between childhood maltreatment, pre-pregnancy BMI status and maternal postpartum mental health. Specifically, we aimed 1) to examine the prevalence of pre-pregnancy BMI categories and different forms of childhood maltreatment in a sample of 741 young adult women who gave birth at the Department of Obstetrics at Leipzig University within a 12-month period; 2) to explore associations between different forms of childhood maltreatment and pre-pregnancy underweight, overweight, and obesity relative to normal weight women taking into account the co-occurrence of other forms of childhood maltreatment, and 3) to examine whether childhood maltreatment accounts for the association between pre-pregnancy BMI and maternal postpartum mental health or whether pre-pregnancy BMI and childhood maltreatment independently predict postpartum mental health.

## Methods

### Procedures

We conducted a cross-sectional study among women who had delivered live-born babies between December 2013 and November 2014 at the Department of Obstetrics (University of Leipzig). The recruitment and data collection took place between February 2014 and March 2015. The study was conducted according to the Declaration of Helsinki and was approved by the Ethical Committee of the University of Leipzig. Participants were only included if they provided written informed consent. Eligible women were identified through an initial review of medical records and contacted within 16 weeks after delivery. If a phone number was available, women were contacted by phone and a study member explained the study verbally to them. Women who verbally agreed to take part in the study were sent written study information material including the questionnaire by postal mail or email, and were asked to provide written informed consent. If a phone number was not available, women were contacted by postal mail containing written study information material and the questionnaire, and were asked to provide written informed consent.

### Participants

A total of 810 women participated in the study and filled out questionnaires. For the current analyses, only women with singleton pregnancies were considered. Therefore, 36 cases with multiple pregnancies were removed from the analyses. Due to missing data with regard to weight and height as well as childhood maltreatment, another 33 cases were removed from the analyses resulting in a final sample of 741 women between 18 and 43 years of age. At the time of the assessment the mean time interval to delivery was 8.10 weeks (SD = 3.15). The majority was married or living with a partner (79.4%) and only 3.0% had a low education. 6.4% reported elevated postpartum depressive symptoms and 20.5% elevated anxiety (Table 1). About 4.6% of the sample reported both elevated postpartum depressive symptoms and elevated anxiety.

**Table 1:** Sociodemographic and postpartum mental health characteristics by pre-pregnancy BMI status

	Total sample *n* = 741	Normal weight *n* = 531	Underweight *n* = 40	Overweight *n* = 114	Obesity *n* = 56	Test statistic	*p*-value
**Demographic characteristics**							
Age, M (SD)	30.58 (4.49)	30.73 (4.42)	29.65 (4.26)	30.79 (4.56)	29.34 (4.98)	2.30^a^	0.076
Nationality other than German, *n* (%)	36 (4.9)	29 (5.5)	0 (0.0)	5 (4.5)	2 (3.6)	2.70^b^	0.440
Married/cohabiting, *n* (%)	588 (79.6)	432 (81.7)	27 (67.5)	88 (77.2)	41 (73.2)	6.80 ^b^	0.079
Multipara, *n* (%)	296 (40.2)	204 (38.6)	14 (35.0)	51 (45.1)	27 (48.2)	3.63^b^	0.305
Low education, *n* (%)	22 (3.0)	11 (2.1)	0 (0.0)	7 (6.1)	4 (7.3)	**10.12** ^b^	**0.018**
**Maternal postpartum mental health**							
Depression (BDI-II ≥ 19), *n* (%)	47 (6.4)	30 (5.6)	2 (5.0)	7 (6.2)	8 (14.3)	6.49^b^	0.090
Anxiety (SCL-90-R anxiety subscale >2, highest quartile), *n* (%)	152 (20.5)	108 (20.3)	8 (20.0)	27 (23.7)	9 (16.1)	1.40^b^	0.706

*BDI* Beck Depression Inventory, *SCL-90-R* Symptom-Checklist-90 revised; ^a^Univariate Analysis of Variance (ANOVA), ^b^Pearson χ^2^ or Fischer’s z-test if applicable; statistically significant differences between pre-pregnancy BMI are printed in bold type

### Measures

*Pre-pregnancy BMI* was calculated from self-reported height and retrospectively reported pre-pregnancy weight (BMI = weight (kg)/height (m)^2^). Pre-pregnancy BMI was categorized according to the WHO into underweight (BMI < 18.5 kg/m^2^), normal weight (18.5 kg/m^2^ ≤ BMI < 25 kg/m^2^), overweight (25 kg/m^2^ ≤ BMI < 30 kg/m^2^) and obesity (BMI ≥ 30 kg/m^2^).

*Childhood abuse and neglect* up to the age of 18 years were retrospectively assessed using the German Version of the Childhood Trauma Questionnaire (CTQ) [41, 42], a 28-item self-report instrument. It covers childhood sexual abuse (“sexual contact or conduct between a child […] and an adult or older person” [41, p. 175]), physical abuse (“bodily assaults on a child […] that posed a risk of or resulted in injury” [41, p. 175]), emotional abuse (“verbal assaults on a child’s sense of worth or well-being” [41, p. 175]), physical neglect (“failure of caretakers to provide for a child’s basic needs” [41, p. 175]), and emotional neglect (“failure of caretakers to meet children’s basic emotional and psychological needs” [41, p. 175]) on a 5-point Likert-scale ranging from *never true* to *very often true.* According to the recommendations by Häuser et al. [39] it was scored into a dichotomous (present vs. absent) and categorical (none, slight, moderate, severe) classification of abuse/neglect. The reliability and validity of the CTQ has been reported. Except for the subscale physical neglect (α = .55), the internal consistency of all subscales was high (α > .80) [43].

*Depression* during the last 14 days prior to the assessment was measured using the German Version of the revised Beck Depression Inventory (BDI-II) [44, 45]. We applied a cutoff of 19 to define cases with at least moderate depressive symptoms. The reliability and validity of the German Version of the BDI-II has been reported [46].

*Anxiety* during the last 7 days was assessed using the anxiety subscale of the German Version of the Symptom Checklist-90-R (SCL-90-R) [47]. As no clinical cutoff score was available we considered women scoring above the 75th percentile as cases with elevated anxiety symptoms. The SCL-90-R has been shown to have good psychometric properties (Cronbach’s α for anxiety subscale = 0.84 [48]).

A variety of *demographic covariates* have been found to be associated with adult BMI [49] and pre- and postnatal mental health [50, 51]. We assessed age, nationality, education, parity, and marital status in a biographical questionnaire as demographic covariates.

### Data analysis

We analyzed data using SPSS, Version 20. Missing values in the demographic covariates were imputed using NORM software and an expectation-maximation algorithm [52]. To answer our research questions, several logistic regression models were conducted. First, associations between childhood maltreatment and pre-pregnancy BMI were examined in multinomial logistic regression models with pre-pregnancy BMI status as dependent variable (reference group: normal weight). Second, associations between childhood maltreatment and postpartum mental health were assessed in logistic regression models using depression and anxiety as dependent variables. Separate analyses for every type of childhood maltreatment (no (reference group) – slight – moderate – severe) were conducted. In a first model, associations were adjusted for demographic covariates. In a second model we additionally adjusted for all other types of childhood maltreatment (present vs. absent). Third, associations between both pre-pregnancy BMI and childhood maltreatment and postpartum mental health were assessed in multivariate logistic regression models using depression and anxiety as dependent variables. In a first step, pre-pregnancy BMI (reference group: normal weight) and demographic covariates were entered as predictors. If pre-pregnancy BMI was significantly associated with postpartum mental health we added different types of childhood maltreatment. In a final step, models were adjusted for all other types of childhood maltreatment (present vs. absent).

## Results

### Prevalence of pre-pregnancy BMI status and childhood maltreatment

**Fig. 1:**
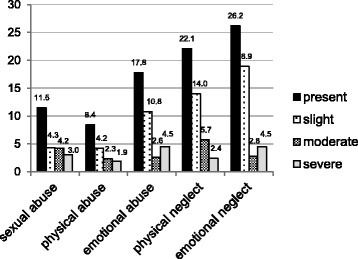
Prevalence of self-reported childhood maltreatment among 741 young adult postpartum women in percent

Five-hundred thirty one women (71.7%) entered pregnancy with normal weight. Fourty (5.4%) entered pregnancy with underweight, 114 (15.4%) with overweight and 5.6 (7.6%) with obesity. We did not find significant overall differences between pre-pregnancy BMI groups for most demographic variables, except for education (*p* = 0.02) with participants with low education being overrepresented in the overweight and obesity categories (Table 1). Three-hundred sixty one (46.0%) women reported any form of childhood maltreatment. One-hundred eighty women (24.3%) experienced one form, 87 (11.7%) two, 28 (3.8%) three, and 31 (4.2%) four different forms of childhood maltreatment. Fifteen women (2.0%) reported all five forms of childhood maltreatment. Emotional neglect was most prevalent (26.2%), followed by physical neglect (22.1%), and emotional abuse (17.8%). Sexual abuse was reported by 11.5% and physical abuse by 8.4% (Fig. 1).

### Childhood maltreatment and pre-pregnancy BMI status

Overall, childhood maltreatment was associated with pre-pregnancy obesity depending on the type and severity of childhood maltreatment (Table 2). In models adjusted for demographic covariates (model 1), severe childhood physical abuse, moderate emotional abuse, and severe physical and emotional neglect were associated with a significantly increased risk of pre-pregnancy obesity (relative to normal weight) (3.33 ≤ OR ≤ 4.43).In model 2, after additionally controlling for all other types of childhood maltreatment, only severe physical abuse was still significantly associated with a 5.2-fold risk of pre-pregnancy obesity (OR = 5.24, 95% CI: 1.15-23.75). Substantial trends for a positive association with pre-pregnancy obesity were still found for moderate emotional abuse and severe emotional neglect (4.07 ≤ OR ≤ 4.20; *p*-values < 0.07). Although increased odds of pre-pregnancy obesity were still observed for severe physical neglect (OR = 3.58, 95% CI: 0.88-14.62) after controlling for the co-occurrence of other types of childhood maltreatment, associations were no longer significant (model 2). Slight physical neglect was associated with a lower risk of pre-pregnancy underweight (OR = 0.14; 95% CI: 0.02-0.94) after controlling for the presence of all other types of childhood maltreatment. Pre-pregnancy overweight was not associated with any type of childhood maltreatment.

**Table 2:** Associations between childhood maltreatment and pre-pregnancy BMI status

	Underweight				Overweight				Obesity			
	Model 1 ^a^		Model 2 ^b^		Model 1 ^a^		Model 2 ^b^		Model 1 ^a^		Model 2 ^b^	
	adj. OR	95% CI	adj. OR	95% CI	adj. OR	95% CI	adj. OR	95% CI	adj. OR	95% CI	adj. OR	95% CI
**Sexual abuse**												
None	*Ref.*		*Ref.*		*Ref.*		*Ref.*		*Ref.*		*Ref.*	
Slight	1.19	0.27-5.34	1.30	0.28-6.04	0.53	0.16-1.81	0.55	0.16-1.89	1.15	0.33-4.03	1.10	0.30-4.02
Moderate	1.79	0.50-6.40	1.61	0.39-6.62	0.46	0.13-1.60	0.35	0.15-1.95	0.33	0.04-2.59	0.23	0.03-1.88
Severe	0.68	0.08-5.48	0.80	0.10-6.74	0.77	0.22-2.74	0.91	0.25-3.37	0.92	0.20-4.28	0.65	0.13-3.19
**Physical abuse**												
None	*Ref.*		*Ref.*		*Ref.*		*Ref.*		*Ref.*		*Ref.*	
Slight	1.32	0.29-5.97	1.80	0.37-8.74	0.58	0.17-1.98	0.63	0.18-2.24	1.45	0.41-5.13	1.75	0.46-6.67
Moderate	4.26	0.82-22.17	5.42	0.82-35.87	0.51	0.10-2.66	0.60	0.12-3.35	2.53	0.59-10.87	3.45	0.70-17.05
Severe	–	–	–	–	0.40	0.05-3.23	0.44	0.05-3.93	**3.86**	**1.10-13.58**	**5.24**	**1.15-23.75**
**Emotional abuse**												
None	*Ref.*		*Ref.*		*Ref.*		*Ref.*		*Ref.*		*Ref.*	
Slight	1.10	0.41-2.95	0.73	0.24-2.24	0.73	0.36-1.47	0.76	0.36-1.61	0.45	1.16-12.94	0.48	0.12-1.62
Moderate	1.27	0.16-10.25	1.15	0.12-11.03	1.13	0.31-4.16	1.36	0.33-5.59	**3.88**	**1.16-12.94**	*4.20*	*0.99-17.86*
Severe	0.57	0.07-4.42	0.45	0.04-4.80	0.48	0.14-1.67	0.71	0.16-3.25	2.08	0.78-5.56	1.90	0.43-8.44
**Physical neglect**												
None	*Ref.*		*Ref.*		*Ref.*		*Ref.*		*Ref.*		*Ref.*	
Slight	0.15	0.02-1.08	**0.13**	**0.02-0.94**	1.11	0.63-1.95	1.13	0.63-2.45	0.79	0.32-1.94	0.77	0.31-1.92
Moderate	0.33	0.04-2.51	0.19	0.01-1.62	0.75	0.29-1.90	0.89	0.33-2.45	1.04	0.34-3.18	0.87	0.24-3.09
Severe	1.45	0.17-12.58	0.76	0.07-7.89	1.02	0.25-4.12	1.45	0.33-6.31	**4.43**	**1.32-14.91**	3.58	0.88-14.62
**Emotional neglect**												
None	*Ref.*		*Ref.*		*Ref.*		*Ref.*		*Ref.*		*Ref.*	
Slight	1.72	0.82-3.61	2.05	0.91-4.63	1.02	0.60-1.73	1.16	0.67-2.01	0.82	0.37-1.82	0.89	0.38-2.08
Moderate	–	–	–	–	1.18	0.38-3.66	2.19	0.60-8.03	0.55	0.07-4.35	0.70	0.07-6.73
Severe	0.75	0.09-5.94	1.68	0.13-22.21	0.91	0.3-2.64	2.34	0.58-9.40	**3.33**	**1.29-8.61**	*4.07*	*0.91-18.35*

Odds ratios from multinominal logistic regression models for pre-pregnancy underweight, overweight and obesity relative to normal weight by different forms of childhood maltreatment (*n* = 741)

^a^adjusted for demographic covariates (age, education, nationality, marital status, parity), ^b^ adjusted for demographic covariates and presence of all other types of childhood maltreatment (present vs. absent)

statistically significant associations (*p* < 0.05) are printed in bold type, substantial trends (*p* < 0.07) are printed in italic type

-- empty cell, Ref. = reference group

### Childhood maltreatment and maternal postpartum mental health

Overall, depending on the severity, all types of childhood maltreatment were positively associated with post-partum depression and anxiety in models adjusted for demographic covariates (Table 3). Postpartum depression was significantly associated with severe childhood sexual abuse and severe physical neglect (7.36 ≤ OR ≤ 9.46). Physical abuse, emotional abuse and emotional neglect were associated with an increased risk of postpartum depression irrespective of the severity (3.13 ≤ OR ≤ 17.36) (model 1). Most associations between childhood maltreatment and depression remained significant after additionally controlling for other types of childhood maltreatment (model 2), except for those of severe physical abuse, slight emotional abuse and physical neglect (any severity grade). In models adjusted for demographic covariates (model 1), physical and emotional abuse, and emotional neglect were associated with an increased risk of postpartum anxiety (2.27 ≤ OR ≤ 7.65) irrespective of the severity. Severe sexual abuse was associated with a 6.7-fold risk of postpartum anxiety and a 2.2 to 4.9-fold risk was found for moderate and severe physical neglect. Severe sexual abuse, slight emotional neglect and all grades of severity of emotional abuse were still associated with a substantially increased risk of postpartum anxiety (1.73 ≤ OR ≤ 5.12) after additionally controlling for all other types of child maltreatment (model 2). All other associations attenuated (Table 3).

**Table 3:** Associations between childhood maltreatment and postpartum depression and anxiety

	Depression (BDI-II ≥ 19)				Anxiety (SCL-90-R anxiety subscale >2, highest quartile)			
	Model 1 ^a^		Model 2 ^b^		Model 1 ^a^		Model 2 ^b^	
	adj. OR	95% CI	adj. OR	95% CI	adj. OR	95% CI	adj. OR	95% CI
**Sexual abuse**								
None	*Ref.*		*Ref.*		*Ref.*		*Ref.*	
Slight	1.03	0.23-4.55	0.53	0.10-2.73	1.42	0.61-3.26	1.01	0.41-2.78
Moderate	–	–	–	–	1.87	0.83-4.20	0.79	0.33-1.93
Severe	**9.46**	**3.62-24.69**	**3.81**	**1.28-11.30**	**6.72**	**2.75-16.41**	**3.70**	**1.42-9.61**
**Physical abuse**								
None	*Ref.*		*Ref.*		*Ref.*		*Ref.*	
Slight	**6.24**	**2.45-15.89**	**3.43**	**1.21-9.77**	**2.63**	**1.23-5.65**	1.44	0.63-3.31
Moderate	**14.93**	**4.75-46.94**	**5.56**	**1.52-20.42**	**4.15**	**1.46-11.79**	1.50	0.49-4.58
Severe	**8.63**	**2.49-29.90**	2.74	0.68-11.00	**3.63**	**1.22-10.83**	1.12	0.34-3.67
**Emotional abuse**								
None	*Ref.*		*Ref.*		*Ref.*		*Ref.*	
Slight	**3.13**	**1.33-7.39**	2.12	0.81-5.45	**3.09**	**1.85-5.16**	**2.46**	**1.42-4.30**
Moderate	**9.32**	**3.03-28.67**	**4.96**	**1.30-18.99**	**7.65**	**2.97-19.70**	**5.12**	**1.81-14.46**
Severe	**17.36**	**7.47-40.38**	**8.28**	**2.28-30.04**	**6.23**	**2.99-12.97**	**3.81**	**1.43-10.16**
**Physical neglect**								
None	*Ref.*		*Ref.*		*Ref.*		*Ref.*	
Slight	1.80	0.82-3.95	1.06	0.43-2.57	1.01	0.60-1.72	0.76	0.43-1.34
Moderate	1.33	0.38-4.68	0.24	0.06-1.0	**2.16**	**1.07-4.34**	0.81	0.36-1.82
Severe	**7.36**	**2.40-22.60**	1.27	0.31-5.16	**4.89**	**1.80-13.28**	1.88	0.61-5.83
**Emotional neglect**								
None	*Ref.*		*Ref.*		*Ref.*		*Ref.*	
Slight	**3.47**	**1.66-7.24**	**2.68**	**1.20-5.98**	**2.27**	**1.48-3.51**	**1.73**	**1.08-2.76**
Moderate	**10.06**	**3.28-30.84**	**4.85**	**1.21-19.39**	**5.02**	**2.05-12.30**	2.01	0.72-5.58
Severe	**14.27**	**5.70-35.77**	**5.59**	**1.39-22.57**	**4.49**	**2.09-9.94**	1.46	0.52-4.10

Odds ratios from logistic regression models for postpartum depression and anxiety by different forms of childhood maltreatment (*n* = 741); *BDI* Beck Depression Inventory, *SCL-90-R* Symptom-Checklist-90 revised; Ref. = reference group; ^a^ adjusted for demographic covariates (age, education, nationality, marital status, parity); ^b^ adjusted for demographic covariates and presence of all other types of childhood maltreatment (present vs. absent); statistically significant associations (*p* < 0.05) are printed in bold type; −- empty cell

### Associations between pre-pregnancy BMI status and childhood maltreatment with maternal postpartum mental health

In the models adjusted for demographic covariates pre-pregnancy obesity was significantly associated with a 2.6-fold increased risk of postpartum depression (OR = 2.55, 95% CI = 1.08-6.00) relative to normal weight (Table 4). No associations were found between anxiety and any pre-pregnancy BMI status Therefore we did not consider anxiety as dependent variable for further analyses.

**Table 4:** Associations between pre-pregnancy BMI status and postpartum depression and anxiety

	Depression (BDI-II ≥ 19)		Anxiety (SCL-90-*R* > 2, highest quartile)	
	adj. OR ^a^	95% CI	adj. OR ^a^	95% CI
**Pre-pregnancy BMI**				
Normal weight	*Ref.*		*Ref.*	
Underweight	0.90	0.21-3.96	1.07	0.48-2.41
Overweight	1.02	0.43-2.40	1.22	0.75-1.99
Obesity	**2.55**	**1.08-6.00**	0.77	0.36-1.64

Odds ratios from logistic regression models for postpartum depression and anxiety by pre-pregnancy BMI status (*n* = 741); BDI-II = Beck Depression Inventory-II; SCL-90-R = Symptom-Checklist-90 revised; ^a^ adjusted for demographic covariates (age, education, nationality, marital status, parity); statistically significant associations (*p* < 0.05) are printed in bold type

Table 5 shows associations of pre-pregnancy BMI and different types of childhood maltreatment with postpartum depression. In the model for pre-pregnancy BMI and sexual abuse, pre-pregnancy obesity and severe sexual abuse independently predicted depression (pre-pregnancy obesity: OR = 2.61, 95%CI = 1.08-6.33; severe sexual abuse: OR = 9.72, 95% CI = 3.69-25.90). In models for all other types of childhood maltreatment (physical abuse, emotional abuse, physical neglect, emotional neglect), the association of pre-pregnancy obesity and postpartum depression attenuated (2.00 ≤ OR ≤ 2.21) to non-significance. When additionally controlling for the presence of all other types of childhood maltreatment, pre-pregnancy obesity was no longer related to depression in any model. In these models, severe sexual abuse (OR = 4.01, 95% CI = 1.35-11.90), slight and moderate physical abuse (3.53 ≤ OR ≤ 5.92), and emotional abuse (4.52 ≤ OR ≤ 8.10), and emotional neglect (all grades of severity) (2.76 ≤ OR ≤ 4.96), were associated with an increased risk of depression.

**Table 5:** Association of pre-pregnancy BMI and self-reported childhood maltreatment with postpartum depression

	Depression (BDI-II ≥ 19)			
	Model 1^a^		Model 2^b^	
	Adj. OR	95% CI	Adj. OR	95% CI
***Pre-pregnancy BMI and sexual abuse***				
**BMI** ^**c**^				
Underweight	1.01	0.22-4.57	0.48	0.09-2.65
Overweight	1.06	0.44-2.54	1.35	0.53-3.41
Obesity	**2.61**	**1.08-6.33**	1.85	0.65-5.25
**Sexual abuse** ^**d**^				
Slight	0.99	0.22-4.46	0.55	0.11-2.88
Moderate	–	–	–	–
Severe	**9.72**	**3.69-25.90**	**4.01**	**1.35-11.90**
***Pre-pregnancy BMI and physical abuse***				
**BMI** ^**c**^				
Underweight	0.69	0.14-3.35	0.59	0.12-3.00
Overweight	1.24	0.51-3.06	1.36	0.54-3.39
Obesity	2.05	0.81-5.22	2.08	0.78-5.59
**Physical abuse** ^**d**^				
Slight	**6.28**	**2.45-16.10**	**3.53**	**1.23-10.13**
Moderate	**15.57**	**4.79-50-70**	**5.92**	**1.55-22.61**
Severe	**7.59**	**2.11-27.31**	2.44	0.59-10.18
***Pre-pregnancy BMI and emotional abuse***				
**BMI** ^**c**^				
Underweight	0.99	0.21-4.62	0.73	0.15-3.52
Overweight	1.27	0.52-3.12	1.35	0.54-3.37
Obesity	2.00	0.75-5.30	1.87	0.68-5.13
**Emotional abuse** ^**d**^				
Slight	**3.27**	**1.38-7.75**	2.22	0.85-5.75
Moderate	**8.47**	**2.70-26.55**	**4.52**	**1.15-17.79**
Severe	**16.92**	**7.18-39.85**	**8.10**	**2.22-29.51**
***Pre-pregnancy BMI and physical neglect***				
**BMI** ^**c**^				
Underweight	0.96	0.21-4.31	0.58	0.12-2.90
Overweight	1.02	0.43-2.43	1.41	0.57-3.47
Obesity	2.21	0.90-5.42	1.92	0.68-5.37
**Physical neglect** ^**d**^				
Slight	1.83	0.83-4.02	0.99	0.40-2.44
Moderate	1.33	0.38-4.67	**0.27**	**0.05-0.87**
Severe	**6.37**	**2.01-20.14**	1.05	0.25-4.52
***Pre-pregnancy BMI and emotional neglect***				
**BMI** ^**c**^				
Underweight	0.94	0.20-4.32	0.70	0.14-3.41
Overweight	1.05	0.43-2.56	1.29	0.52-3.25
Obesity	2.10	0.81-5.39	1.96	0.73-5.32
**Emotional neglect** ^**d**^				
Slight	**3.51**	**1.67-7.36**	**2.76**	**1.22-6.10**
Moderate	**10.52**	**3.40-32.57**	**4.80**	**1.20-19.22**
Severe	**12.66**	**4.96-32.34**	**4.96**	**1.21-20.30**

Results from logistic regression models (*n* = 741); BDI-II = Beck Depression Inventory-II; ^a^ adjusted for demographic covariates (age, education, nationality, marital status, parity); ^b^ adjusted for demographic covariates and presence of all other types of childhood maltreatment (present vs. absent); ^c^ reference group: normal weight; ^d^ reference group: none; statistically significant associations (*p* < 0.05) are printed in bold type; −- empty cell

## Discussion

Our study aimed to contribute to a better understanding of the role of childhood maltreatment in pre-pregnancy obesity and postpartum anxiety and depression: two common and interrelated conditions affecting the mother’s and the child’s health during pregnancy and postpartum. Even though maternal obesity is a very unique type of adult obesity, due to the particular health risks associated with it [1–4], and the perinatal period can be seen as a period of increased vulnerability to psychological distress, associations between the two have been addressed very little [16]. The role of childhood maltreatment in this association has not been explored thus far.

In our sample almost 8% of included women entered pregnancy with obesity. 46% reported a history of any abuse or neglect during childhood, with emotional and physical neglect and emotional abuse being reported most frequently. Considering associations between histories of childhood maltreatment and maternal obesity, we found that severe childhood physical abuse, moderate childhood emotional abuse and severe physical and emotional neglect were independently associated with a higher risk of pre-pregnancy obesity relative to normal weight. Even after adjusting for the co-occurrence of all other forms of childhood maltreatment, women with severe childhood physical abuse had a fivefold likelihood of pre-pregnancy obesity. A new finding of our study was that, the relationship between severe childhood physical abuse and pre-pregnancy obesity was even stronger after controlling for the co-occurrence of other types of childhood maltreatment. This result underpins the importance of childhood physical abuse in pre-pregnancy obesity. Considering maternal postpartum mental health, our results indicate that all forms of childhood maltreatment, depending on the severity, increased the risk of depression and anxiety after delivery. Pre-pregnancy obesity was associated with a 2.55-fold increased risk of postpartum depression but was not associated with anxiety. After including both, different forms of childhood maltreatment and maternal obesity, in multivariate models predicting postpartum depression, we found that severe childhood sexual abuse and maternal obesity independently predicted postpartum depression. In models for other forms of childhood maltreatment associations between pre-pregnancy obesity and depression diminished to non-significance while postpartum depression was still significantly associated with histories of childhood physical and emotional abuse as well as physical and emotional neglect. These results can be considered as an indication that – with the exception of childhood sexual abuse – childhood maltreatment may at least partly account for the association between maternal obesity and postpartum depression.

Our results reflect some findings from previous studies. The prevalence of maternal obesity in our sample was lower compared to studies conducted in US populations [1] but comparable to the prevalence reported in community-based European studies [1, 4]. A number of general population studies suggest that childhood maltreatment is associated with a higher risk of adult obesity and anxiety, and both childhood maltreatment and obesity are associated with depression [11, 12, 24, 26, 28]. While studies from the general population suggest an association between sexual, physical and emotional abuse and adult obesity [26] only severe physical abuse was significantly associated with pre-pregnancy obesity in our study. Furthermore, the results from our study are in line with findings from a recent meta-analysis, suggesting that women who entered pregnancy with obesity are at higher risk of ante- and postnatal depression [16], which has been shown to negatively impact on the child’s development [19–21]. Women with low education were significantly overrepresented in the overweight and obesity categories in our study, a result which corresponds with findings from the general population [53]. Low education has also been found to be a significant predictor of pre-pregnancy obesity and associated risks [54]. Hemmingsson [53] argues, that emotional distress may serve as an important mediator in the association between socioeconomic disadvantage and adult obesity. It is important to note, however, that the above mentioned associations were adjusted for the educational level. A new finding of our study was that when different forms of childhood maltreatment and maternal obesity were considered simultaneously in a multivariate model, maternal obesity was no longer predictive of postpartum depression. The only exception was childhood sexual abuse and maternal obesity, which were found to independently predict postpartum depression. This finding leads us to the conclusion that both maternal obesity and postpartum depression may be outcomes of childhood maltreatment and that childhood maltreatment may at least partly contribute to the association between pre-pregnancy obesity and postpartum depression. Despite the cross-sectional and retrospective nature of our study, the hypothesis that both maternal obesity and postpartum depression share common mechanisms which could be triggered through severe childhood maltreatment becomes plausible, when looking at neurophysiological and psychological long-term changes as a consequence of childhood maltreatment. There is evidence for elevated inflammation levels in maltreated individuals [55–58], which has also been found being associated with depression and obesity [10, 11]. Furthermore, childhood maltreatment may lead to a chronic dysregulation in physiological stress-regulation systems (e.g. HPA-axis) which has been found to be involved in obesity and depression [10, 11]. Common mechanisms may also lie in psychological or psychosocial consequences of childhood maltreatment, e.g., psychosocial disadvantages, social isolation, low SES, and stigma [53, 58, 59]. However, the magnitude of the associations between maternal obesity and postpartum depression was still slightly increased in some models (1.85 ≤ OR ≤ 2.08). A lack of power may have been the reason that these associations failed to reach statistical significance. To clarify the question whether childhood maltreatment fully accounts for the association between pre-pregnancy obesity and depression, it would be necessary to test these associations in larger samples with higher base rates of pre-pregnancy obesity.

Before discussing the strengths and limitations of our study in detail, it is important to point out that our study represents a cross-sectional snapshot focusing on two factors (maternal obesity, postpartum depression) related to childhood maltreatment which have to be interpreted in the light of a complex cycle of factors leading to long-term maladaptation as a consequence of early childhood maltreatment. Childhood maltreatment could be seen as a risk factor in the development triggering a chain of further risks which are associated with physical and mental health problems in later life [12], including trauma and PTSD in adulthood, intimate partner violence and parenting problems amongst others [30, 40, 60, 61]. Several mediating pathways between histories of childhood maltreatment, perinatal mental health and parenting problems have been proposed (e.g., [60, 62]); something which we were not able to account for in our study. Nevertheless, our study adds to this debate by focusing on long-term health consequences of childhood maltreatment among pregnant and postpartum women, which may bear health risks for the mother and the child.

A major limitation of our study is the cross-sectional design precluding any conclusions on causality. Furthermore, no information on the exact timing regarding the onset and ending of childhood maltreatment, the onset of obesity or lifetime depression history was available in our study. Taking into account results from prospective general population studies on the effects of childhood maltreatment on weight gain from childhood into adulthood [31–33] it seems plausible to assume that obesity starts long before postpartum depression. However, as no information on mental health before or during pregnancy was available it remains unclear whether childhood maltreatment and pre-pregnancy obesity increase the risk of incident postpartum depression as a special entity of depression or of depression in adulthood in general. Childhood maltreatment was assessed retrospectively and relied on the recall of adverse childhood experiences which is likely associated with measurement error [63]. Objectively measured height and pre-pregnancy weight would have been preferable. Although self-reported and objectively measured weight during pregnancy are strongly correlated [64], self-reports might be subject to recall error. Although the sample size in our study is quite large compared to similar studies among pregnant women [34] it might have been underpowered to detect further associations between pre-pregnancy BMI (particularly underweight) and childhood maltreatment or postpartum mental health due to the relatively low rate of women with pre-pregnancy obesity. A further limitation of the study refers to the limited generalizability of the findings, particularly to women with lower education levels. Despite the large sample size only 3% of our sample had a low education.

A major strength of our study is that we took the likely co-occurrence of several forms of childhood maltreatment into account and considered different grades of severity of childhood maltreatment and the whole BMI spectrum, allowing for a more precise evaluation of the association between childhood maltreatment and pre-pregnancy BMI relative to normal weight.

## Conclusions

The results of our study suggest that childhood maltreatment is associated with two risk obstetric conditions, pre-pregnancy obesity and postpartum depression and anxiety, each of them bearing several health risks for the mother and the child. Therefore our results underline the potential long-term health consequences of early traumatic experiences during childhood. Thus routine screening of childhood maltreatment history is warranted in prenatal care to identify women at risk. A history of abuse and neglect may be an important barrier to both, effective obesity and depression/anxiety treatment. Furthermore, our results suggest that pregnant women with obesity may be particularly vulnerable to postpartum depression and more likely to have experienced childhood maltreatment. Clinicians should be aware of these associations to provide targeted counselling. Prospective studies are needed to clarify the mechanisms of these associations. Furthermore, future studies should take into account further factors which might play a role as possible mediators in these associations, e.g., intimate partner violence or traumatic experiences over the life-span.

## Abbreviations

BDI: Beck depression inventory
BMI: Body mass index
CTQ: Childhood trauma questionnaire
SCL-90-R: Symptom-Checklist-90 revised
